# Consultations through the Journal

**Published:** 1841-11

**Authors:** 


### CONSULTATIONS THROUGH THE JOURNAL.

While writing the above, it occured to us, that many consultations and much reciprocal instruction might be conducted, through our monthly. We refer of course to chronic diseases. Cases are constantly occuring of an eccentric or anomalous character, whlch puzzle the medical attendant, and naturally turn his mind towards his professional cotemporaries. All such might be stated in the Journal, the readers of which could not fail to peruse them with interest, and whoever could, would no doubt bring to bear upon them, the results of his own experience. In this way much fresh and valuable information might be extensively communicated. Whenever cases are thus submitted, they should be drawn up with care. Fulness, clearness and brevity should characterize them; and while nothing of moment ought to be omitted, nothing unimportant shóuld be introduced. All communications thus prepared will be promptly inserted. They must be subscribed by their authors, but the names will not be published unless requested. D.

